# Genetic Monitoring of Grey Wolves in Latvia Shows Adverse Reproductive and Social Consequences of Hunting

**DOI:** 10.3390/biology12091255

**Published:** 2023-09-19

**Authors:** Agrita Žunna, Dainis Edgars Ruņģis, Jānis Ozoliņš, Alda Stepanova, Gundega Done

**Affiliations:** Latvian State Forest Research Institute Silava, Rīgas Str. 111, LV-2169 Salaspils, Latvia; dainis.rungis@silava.lv (D.E.R.);

**Keywords:** wolves, kinship analyses, social structure, restricted hunting

## Abstract

**Simple Summary:**

Genetic indicators of animal populations are an important part of the parameters used to describe the population status. In addition, for social species, such as the grey wolf, the preservation of typical kinship relations and social structure is important to ensure the existence of populations as a well-functioning part of an ecosystem. Hunting can affect the genetic parameters and the stability of social structures. In this study, muscle tissue samples from wolves hunted in Latvia were collected between 2009 and 2021 for genetic and kinship analyses. It was established that the hunting pressure during this time had not caused negative changes in the genetic parameters of the population. The typical pack structure was observed; however, negative consequences of hunting were found in breeder loss, pack disruption, a loss of pack territories, and the early dispersal of juveniles. Although similar processes also occur in unexploited wolf populations, they affect the social structure of the population in less disruptive ways than the impact of high hunting pressure. Therefore, the impact of hunting on the kinship and social structure of the population should be further monitored and the consequences that such disturbances may have on the conservation of the population should be clarified.

**Abstract:**

Nowadays, genetic research methods play an important role in animal population studies. Since 2009, genetic material from Latvian wolf specimens obtained through hunting has been systematically gathered. This study, spanning until 2021, scrutinizes the consequences of regulated wolf hunting on population genetic metrics, kinship dynamics, and social organization. We employed 16 autosomal microsatellites to investigate relationships between full siblings and parent–offspring pairs. Our analysis encompassed expected and observed heterozygosity, inbreeding coefficients, allelic diversity, genetic distance and differentiation, mean pairwise relatedness, and the number of migrants per generation. The Latvian wolf population demonstrated robust genetic diversity with minimal inbreeding, maintaining stable allelic diversity and high heterozygosity over time and it is not fragmented. Our findings reveal the persistence of conventional wolf pack structures and enduring kinship groups. However, the study also underscores the adverse effects of intensified hunting pressure, leading to breeder loss, pack disruption, territorial displacement, and the premature dispersal of juvenile wolves.

## 1. Introduction

In the last two decades, the use of molecular genetic methods has become widespread in studies of animal species and in addressing conservation issues [1,2]. The collection and analysis of genetic material are standard procedures in many large carnivore monitoring programs. In wolf research, genetic methods are used to assess population distribution, numbers, the size of packs and their territories [3,4], to characterize dispersal habits [5,6,7,8], to detect breeding [4,9], to recognize individuals and determine kinship structure [9,10,11,12,13], to study population genetic parameters, phylogenetic, and systematic issues [10,14,15], and to detect hybridization between different species [16,17,18]. As the grey wolf is a protected species in many countries, genetic material is mostly obtained noninvasively [1,3,6,15,19,20], and less often from legally or illegally hunted or accidentally killed animals [3,12,21,22,23]. Information obtained from genetic analyses is important for management decisions in small, endangered, and isolated populations to ensure their survival [21,24]. It is also relevant in exploited populations, where such research can provide information on the effects of hunting on the genetic and social structure of the populations [11,12]. In Latvia, DNA samples from hunted wolves have been used in studies of the genetic diversity of the population, phylogenetic processes, and hybridization with dogs [17,18,25,26,27].

In Latvia, the wolf is a specially protected species of limited use. Before Latvia’s accession to the European Union and the implementation of the Habitats Directive 92/43/EEC of the Council of Europe, wolves were hunted without any restrictions, and at the beginning of the 2000s, the number of predators significantly decreased. In 2000, the development of the first wolf species conservation plan was started in Latvia [28], and since 2004, an annual quota has been set and a wolf hunting season has been established. After the introduction of restrictions, the number of wolves in the population gradually increased [29,30]. The goal of the protection and management of the species in Latvia, according to the current conservation plan, is to preserve the favourable conservation status of the grey wolf population for an unlimited period of time and to promote the maintenance of this status [29]. One of the objectives of species conservation is to ensure biological diversity, and this also includes the maintenance of favourable genetic parameters and the social structure of populations. According to the criteria of the International Union for Conservation of Nature, the Latvian wolf population is assessed as “least concern” [31], but it is subject to relatively high hunting pressure [29,30], and periodic hybridization between wolves and dogs has also been detected [17,18]. The hunting pressure on the Latvian wolf population is higher than that on the wolf populations in Estonia and Lithuania [32,33], but a similar amount of population control is implemented in neighbouring Belarus [34]. Intensive wolf harvest can be numerically sustainable as wolf populations can withstand a relatively high hunting mortality (29–60%) without a decrease in the number of animals [35,36,37,38], especially if the impact of hunting is mitigated by the immigration of individuals from neighbouring populations and/or abundant feeding conditions that favour high reproduction [36]. However, high hunting pressure can still have a negative impact on the demographic, social, and genetic structures of the population [11,12,39,40].

Wolves mostly live in packs consisting of related animals—a pair of breeding animals and their offspring [41,42]. It is considered that social living in families or groups improves the adaptation of individuals for several animal species, increasing hunting, territorial defence, and reproduction success, promotes the survival of offspring, helps to resolve conflicts, and altruistic behaviour is also more often observed among related individuals [43,44,45]. Human-caused mortality can lead to breeder turnover, pack dissolution, an abandonment of pack territory or a reduction in pack size [46,47,48], as well as inbreeding among pack members or hybridization with other species [11,24,49]. A reduced degree of relatedness within packs is also reported [50]. Packs and their stability are the basic unit of the wolf population, and their conservation should be considered when planning wolf population management measures. The disruption of wolf packs, for example by hunting, affects both the social and genetic structure of the population. Changes in the social structure of wolf populations can have negative consequences for the demographic parameters of the population and lead to changes in animal behaviour, hunting, breeding and dispersal habits, and gene flow between packs, which can then lead to changes in a population’s genetic structure [3,12,24,37,47,51]. Subsequently, the changes in the genetic structure can contribute to changes in the genetic diversity parameters of the population, an increase in the degree of inbreeding, and an associated decrease in the adaptability and evolutionary adaptations of individuals and the whole population [2,3,21,50,52]. Inbreeding and low genetic diversity can reduce reproductive success, increase mortality, promote the expression of deleterious alleles, increase susceptibility to various parasites and pathogens, and reduce the species’ adaptability to new diseases, toxins, environmental conditions, and climate change [2,21,40,53]. Such effects may initially appear to be minor, but over a longer period of time, they can have serious consequences affecting the sustainable existence of the populations and the conservation of the species [53]. In species living in social groups with limited breeding opportunities, hunting can have complex effects on a population’s social and genetic structures [49,54]. Hunting can reduce the genetic diversity of a population by reducing the exchange of individuals between populations or its units, or in some cases, it can increase genetic diversity as it can cause social instability, higher dispersal rates, and the immigration of individuals from neighbouring populations. Therefore, to ensure the successful conservation of the species, it is necessary to assess the impact of hunting on various parameters characterizing the status of the population, including the social and genetic structure of populations [12,55]. As complete knowledge as possible about the status of wolf populations is required, including the processes occurring within them and the impact of human activities. Genetic monitoring data should be taken into account in the management of hunted species [10,52,56], as they sometimes can reveal that population status is not as favourable as the census and distribution data would suggest [57].

The aim of this study is to describe several genetic characteristics and the kinship structure of the restrictedly harvested wolf population in Latvia and to assess the effect of current hunting practices on the genetic parameters, social and territorial structure, and the possibilities for the sustainable conservation of the population.

## 2. Materials and Methods

### 2.1. Study Area and Wolf Harvest in Latvia

Material and data for this study were collected throughout the entire territory of Latvia, which occupies 64.6 thousand km^2^ along the eastern coast of the Baltic Sea. Wolves are distributed and harvested in the whole country with the exception of the territory around the capital Riga. Approximately 50% of the region is covered with mainly mixed boreal forests. A hunting season from 15 July to 31 March and an annual hunting quota were introduced in Latvia in 2004. The quota is set annually for the whole country. It has been increasing since 2004 and was around 270–300 animals for the last ten hunting seasons. Official census data in the last decade have estimated the number of wolves in Latvia to be around 1200 individuals, while Virtual Population Analyses estimates of minimum wolf abundance for the same period were around 700–800 individuals. Hunting mortality was estimated to be about 37% in the last two decades [30]. Wolves are hunted both during specially organized hunts and also during hunts of other game animals, and wolf hunting in Latvia can be considered nonselective. The main prey species for wolves are wild ungulates, mostly roe deer (*Capreolus capreolus*) and wild boar (*Sus scrofa*), and prey availability during the study period was not a limiting factor for the population [29].

### 2.2. Sample Collection and Analyses

Data and material were gathered from legally hunted or otherwise killed (traffic accidents and scabies) wolves throughout the country in the framework of an ongoing wolf monitoring program. For genetic analyses, we collected 1363 muscle tissue samples between 15 July 2009 and 31 March 2021. A total of 1269 individuals (662 males and 607 females) were successfully genotyped (Table S1) accounting for 42.4% of all wolves harvested during this period. A summary of the sample distribution per year is shown in Table S2. We collected tooth samples from 985 animals to determine the exact age by the number of growth lines in the dental cementum [58]. Birth year was calculated from the determined age, and 862 individuals with a known birth year were included in the analysis.

The date and location of hunting were known for each hunted wolf. Since 2015, the coordinates of the hunting location had been marked, and prior to that, the parish where the individual was hunted. We calculated straight line distances of movements of related individuals between centres of parishes where animals were harvested.

Muscle tissue samples collected from animals for genetic analyses were stored at −20 °C. DNA was isolated from approximately 30 mg of muscle tissue using the E.Z.N.A Tissue DNA Isolation Kit (Omega Bio-Tek/VWR, Norcross, GA, USA). A total of 16 autosomal microsatellite loci that were previously used in analyses of Latvian and Estonian wolf populations [26] were genotyped: FH2001, FH2010, FH2017, FH2054, FH2079, FH2088, FH2096 [59], vWF [60], AHT130 [61], M-CPH2, M-CPH4, MCPH12 [62] and C09.173, C466, C20.253, and CXX225 [63]. The forward primer was synthesized with a 6-FAM, HEX, or TMR fluorescent label to allow the visualisation of amplification products on a genetic analyser. Multiplex polymerase chain reactions (PCRs) were performed in a total reaction volume of 20 μL containing ca. 50 ng DNA, 4 μL of 5× HOT FirePol^®^ Blend Master Mix (Solis BioDyne, Tartu, Estonia, final magnesium chloride (MgCl_2_) concentration of 2 mM), and 0.2 μM of forward (labelled) and reverse primers. PCR conditions were as follows: 95 °C for 15 min and 30 cycles of 95 °C—30 s, 58 °C—40 s, 72 °C—60 s, and 72 °C—10 min. PCR products were separated on an ABI 3130xl Genetic Analyzer (Applied Biosystems, Waltham, MA, USA) and genotyped using GeneMapper 4.0.

The genetic parameters of the population (observed heterozygosity, expected heterozygosity, inbreeding coefficient (fixation index), Nei genetic distance, population differentiation (Fst), mean pairwise relatedness, and number of migrants per generation (Nm)) and PCoA based on pairwise Nei genetic distances were calculated using the standard parameters in the GenAlEx 6.5 program [64]. Indices of mean pairwise relatedness were calculated using the QGM [65] estimator using GenAlEx 6.5. Allelic diversity (richness) was calculated using Fstat 1.2 [66]. Kinship analyses were performed using the COLONY 2.0.5 [67] and CERVUS [68] programs. For pedigree analyses, male and female polygamy was allowed, and no inbreeding was assumed. Allelic dropout and other genotyping errors (including mutations) were set at 0.001 and 0.005 for each marker, respectively. We analysed full sibling relationships and parent–offspring relationships. The entire sample was analysed for full sibling relationships, regardless of the known age of the animals. In addition, the sample was divided according to the year of birth of the animals, which was calculated taking into account the known age of the animals. We used these data in parent–offspring analyses, assuming that the potential parents were at least two years older than the offspring. Individuals were assigned to kin groups (as sibling or parent–offspring relations) if probability of kinship was above 0.95. However, in some cases where juvenile animals were harvested at the same date and location, lower probability levels were accepted for kinship assignment. Kinship analyses were used to construct kin groups, incorporating additional data about age, sex, and harvesting date of individuals.

**Figure 1:**
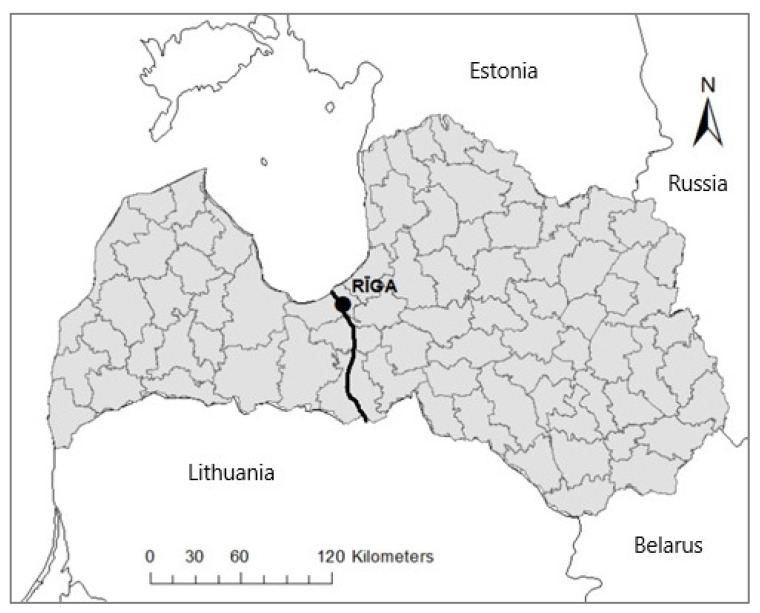
Samples for genetic analyses were collected from 2009 to 2021 throughout the entire territory of Latvia. Western (*n* = 506) and eastern (*n* = 763) regions were defined (the line in the centre of the country indicates the region boundary).

The samples were divided into two groups according to the geographical location of their collection in order to compare genetic parameters between the western (*n* = 506) and eastern (*n* = 763) parts of the populations (Figure 1) to examine if the wolf population of Latvia could have split into two subpopulations. Such fragmentation has been considered as a possible threat due to the urban and agricultural landscape of the central part of the country that could hinder the migration of the animals between the eastern and western regions [69]. For analysis, the samples were also divided according to the birth year of individuals.

## 3. Results

### 3.1. Population’s Genetic Characteristics

During the study period from 2009 to 2021, the average expected heterozygosity (He) of the entire population was 0.732 ± 0.018, the observed heterozygosity (Ho) was 0.713 ± 0.018, and the inbreeding coefficient (F) was 0.026 ± 0.006. The grouping of individuals according to birth year indicated that the groups were not genetically differentiated (overall Fst = 0.004, *p* = 0.001), and the genetic diversity parameters were similar (Figure S2.1). The allelic diversity ranged from 5.81 to 6.39 and was not significantly different among the birth year groups (F(13, 210) = 0.24, *p* = 0.997). During this time period, there was a significant tendency for the observed heterozygosity to increase (F(1, 11) = 23.47, *p* = 0.001, R^2^ = 0.68) and the inbreeding coefficient to decrease accordingly (F(1, 11) = 22.22, *p* =0.001, R^2^ = 0.67) (Figures S2.2 and S2.3).

**Figure 2:**
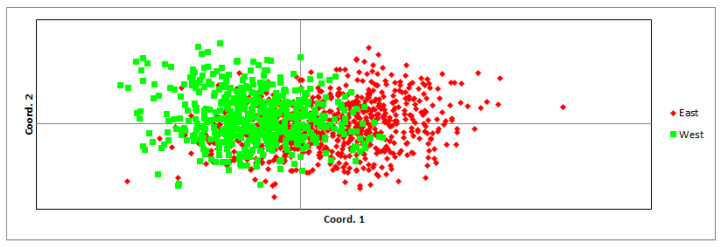
Principal Coordinates (PCoA) based on pairwise Nei genetic distances between individuals harvested in the eastern and western regions; data were collected from 2009 to 2021.

Comparing the eastern and western parts of the populations, the average expected and observed heterozygosity did not differ significantly: 0.737 ± 0.019 and 0.722 ± 0.020 in the east and 0.706 ± 0.018 and 0.700 ± 0.022 in the west, respectively. Allelic diversity was also not significantly different between both parts of the population (in the east—8.15 and in the west—7.74; t(30) = 0.51, *p* = 0.611). Comparing the genetic distance between individuals in both regions, some differentiation was observed (Figure 2), but the eastern and western parts of the populations were not genetically differentiated (Fst = 0.019, *p* = 0.001). Inbreeding coefficients in both parts of the populations were low: 0.020 ± 0.008 in the east and 0.010 ± 0.008 in the west. Thirteen migrants per generation were estimated between the eastern and western regions. Significant differences in mean pairwise relatedness were found: in the western region, it was higher (0.048 ± 0.001) than in the eastern region (−0.001 ± 0.001).

### 3.2. Population Kinship Structure and Pack Dynamics

During the study period, 223 groups of related wolves were determined based on full sibling and parent–offspring relationship analyses, incorporating the age, sex, and harvesting date of individuals. The smallest groups consisted of 2 animals, and the largest group consisted of 15 animals. These kin groups included animals from one or more wolf packs and individuals of the same group could be hunted in different hunting seasons. In the majority of groups, related individuals were found only during one or two hunting seasons (46.1% and 15.6% of all groups, respectively). Less than a third of the groups existed for four years or longer (29.4%) (Table 1). The longest time period over which related animals within the same kin group were hunted was 11 years. Most of the groups that existed for four years or longer were hunted in the western (47.5%) and northern parts of Latvia (26.2%). Fewer such groups were found in the southeast (14.8%) and the south (11.5%). No groups that had existed for seven years or more were found in the eastern part of Latvia. We detected the migration of individuals between the eastern and western parts of Latvia in 19 kin groups.

**Figure 3:**
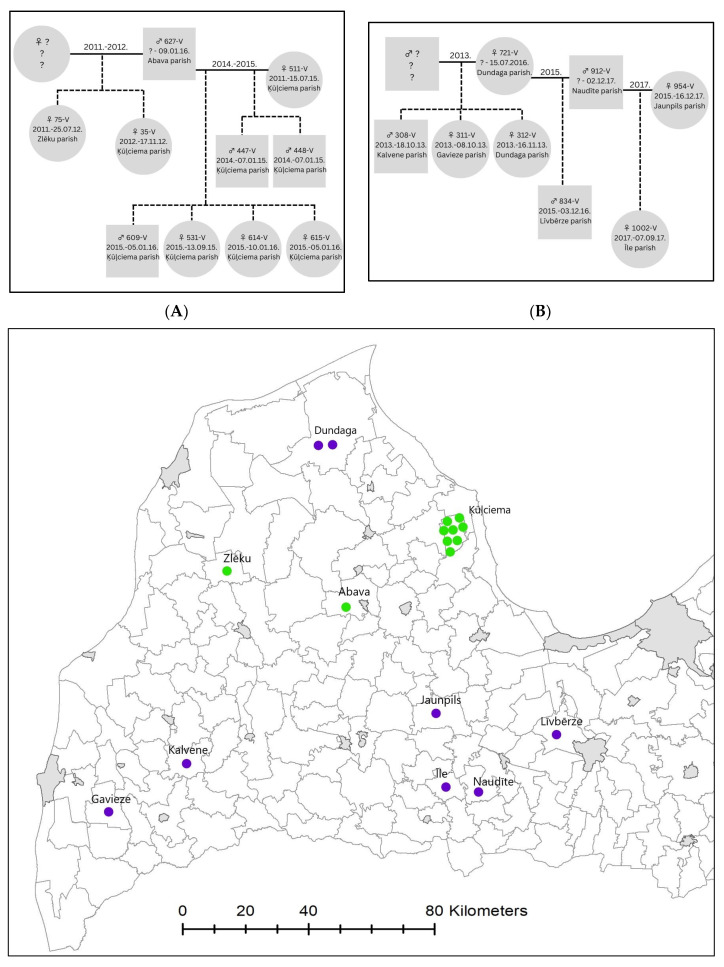
Relationships in two wolf packs in the western part of Latvia and hunting locations of the individuals (pack (**A**) in green; pack (**B**) in purple). Continuous lines mark breeding pairs; years above the line indicate estimated time period of breeding pair relationship. Dotted lines mark offspring. Number of the sample, year of birth, and date and place of harvest are shown for every wolf. The “?” marks unknown individuals that were not sampled or indicates information about the sampled individuals was missing.

We detected the loss of at least one breeder in 64.6% of the identified kin groups. In 27.1% of the cases, the breeder was hunted while at least one or more pups were still alive. In 17.5% of the cases, one of the breeders was hunted while pups were three to ten months old (5.6 ± 2.0 months old on average). In some packs, we could trace the loss of the breeder and the replacement of the lost individual over the years; two examples are shown in Figure 3. In four packs, after one of the breeders and at least some of the other pack members were hunted, the other breeder was hunted one to two years later in a relatively distant area (straight line distance of 35, 40 (Figure 3, pack A), 45, and 110 km (Figure 3, pack B)), indicating the abandonment of the original pack territory. In three more families, one of the breeders moved within six months to five years and was hunted 30, 35, and 90 km away from the original pack territory where his pups were hunted, but in these cases, we did not know what happened to the other breeder.

Out of 39 packs in which the loss of one or both of the breeders was detected, the juveniles were hunted during the current hunting season within the same parish in 13 packs and within the boundaries of neighbouring parishes in 17 packs, and the dispersal of juvenile animals beyond 30 km was observed in 9 packs. It should be noted that according to genetic data, in cases where one of the breeders was lost, we did not always know what happened to the other breeder. Perhaps the young animals left the natal territory together with the surviving pack members, but it is also possible that juveniles left the natal territory on their own. In six families, after the loss of one of the parents, the juvenile animals remained with the other parent in the same or neighbouring parish where they were hunted later during the same hunting season. In nine families where the loss of one breeder was confirmed, the juvenile animals were hunted later in the same hunting season 30 to 230 km away, indicating that the juveniles had left their natal territories at a relatively young age.

## 4. Discussion

The genetic parameters obtained in this study indicate that, overall, the Latvian wolf population is in a favourable status—inbreeding is low, allelic diversity has not decreased over the years, and genetic diversity is high and similar to that found in Estonian [26], Polish [50], Lithuanian [70], and some North American populations [10,11,12,24]. Heterozygosity had a tendency to increase, while the inbreeding coefficient had a tendency to decrease during the study period. Although, in general, such trends can be evaluated as favourable, they also indicate a relatively high turnover of individuals in the population. This occurs in Latvia as a result of hunting and the subsequent arrival of immigrants from other parts of the population within the country or from neighbouring countries [50,71]. The short duration of more than half of the established kin groups (Table 1) also indicates a high turnover of individuals and whole packs in the population, which contributes to the observed increase in heterozygosity.

In earlier years [69], there were concerns that the wolf population of Latvia could split into two subpopulations, due to the hindered migration of animals across the central part of the country due to the agricultural and urban environment of the territory. However, the results of genetic (Figure 2) and kinship analysis showed that animal migration between the two parts of the country is not hindered and the western and eastern parts of the population are not isolated. Genetic distance, heterozygosity, and inbreeding rates were not significantly different between both parts of the population. A sufficient number of migrants per generation to maintain genetic diversity [2] was detected between the western and eastern regions. However, the assessment of genetic parameters may not provide complete information about the current migration between parts of the population, as low genetic divergence may also be a consequence of relatively recent historical migration [72]. Therefore, we also used kinship analyses to determine the migrations of individuals across the central part of Latvia, and they confirmed the results of genetic parameter analyses by identifying the migrations of related animals from 19 kin groups.

Although the western and eastern parts of the Latvian population are not isolated, we observed higher mean pairwise relatedness in the western part of the population compared to the eastern part. This suggests a turnover of individuals between nearby packs in the west, thus ensuring higher relatedness among packs, while in the east, nonrelated animals dispersing further from their natal packs or immigrating from the territories of neighbouring countries might be accepted into packs more often [69]. The relatedness within the population and the self-sustaining capacity of the population can be judged by the duration of the existence of the kin groups. A frequent and rapid replacement of kin groups indicates disrupted breeding pairs, where lost animals are replaced by unrelated individuals arriving from neighbouring territories [3,42,50]. The unstable social structure and a lower degree of relatedness among packs characteristic of exploited populations [10,51,73] create favourable conditions for the acceptance of foreign animals into the packs. The rapid acceptance of strangers in place of lost individuals is beneficial for the preservation of the pack, ensuring reproduction and reducing inbreeding [35]. The longest existing and geographically closest groups were found in the north regions of the country, while the least long-standing kin groups were found more in the eastern and central parts of Latvia. This indicates that hunted individuals in these areas are quickly replaced by immigrants from further regions of the population within the country or parts of the population in neighbouring countries, thus reducing relatedness both within packs and among packs and leading to the detected differences in relatedness between the eastern and western regions. Better reproductive success in wolves and higher resistance to the effects of hunting in Western Latvia, compared to the southeast and northeast regions, was also found in an earlier study [69]. These observations can be at least partially explained by the closer kinship of the wolf population and the greater stability of the social structure in the western part of the country, which accordingly improves the chances of the sustainable existence of this part of the population, whereas long-term maintenance of the population relying on immigrants from neighbouring areas, as is probably occurring to some extent in Eastern Latvia, can be a risky and unstable strategy in case of a decline in the source population(s) or if some obstacles emerge that hinder animal immigration, such as fences built along country borders [31].

In groups where a sufficient number of genetic samples were collected, we observed the typical structure of wolf packs—a breeding pair and their offspring of two years [41,42]. The loss of a breeder was often detected (64.6%) and, in some cases, it was possible to trace the replacement of a partner within a pack (Figure 3). A negative effect of hunting on the social structure of the wolf population was observed. Significant numbers of packs lost one or even both of the breeders during one hunting season, which could then affect the survival chances of pups. The average age at which pups lost at least one of the parents was 5.6 months. Although pups are able to accompany adult animals on a hunt to learn hunting skills at the age of four months [74] and they start to catch small animals at a relatively young age, they still depend on the help and training of the adult pack members until the age of nine to ten months [41,74]. At least some pups in the litter can survive the early loss of a parent due to other adult members of the pack continuing to provide for them; however, the loss of even one parent can make it difficult to take care of pups, and can affect the social and spatial structure of the pack. As a result of pack disruption, depredation on livestock by inexperienced young animals who have lost their parents or have dispersed from their natal packs at an early age can occur [47,49,51,75]. In order to reduce such a negative impact on the composition of wolf packs and young animals, it is recommended to begin wolf hunting only when the juveniles have reached at least six months of age [47]. In the case of Latvia, this would mean shortening the hunting season and beginning hunting only in October–November. Currently, Latvia has the longest wolf hunting season in the Baltic States (15 July–31 March), while in Estonia, the wolf hunting season lasts from 1 November to the end of February [33], and in Lithuania, from 15 October to 1 April [76].

The loss of a breeder also affects the persistence of the pack and maintenance of the pack’s territory. The fact that in four packs, after the loss of a partner, the other breeder was hunted at a relatively distant location within one to two years suggests that due to hunting disturbance, the surviving breeder left the previous territory and searched for a new partner and territory. We also detected the dispersal of subordinate animals after the loss of the breeders. The territories of wolf packs are quite stable, and as long as one of the breeders and some other animals of the pack have been able to survive, the pack usually maintains its territory [3]. In saturated populations, subordinate animals will usually stay with the natal pack even after the loss and replacement of the breeder as their own breeding options are limited due to the lack of vacant breeding positions [42]. Wolf density and numbers in Latvia are currently high [30]; therefore, pack disruption, the dispersal of young animals, and the abandonment of the territory by breeders indicate the negative impact of a sufficiently large disturbance on the stability of the population’s social structure. Subordinate individuals disperse when breeding opportunities are easily found, as happened in a population affected by high harvest mortality [48]. Although cases where information about further processes in the pack was available were numerically few in this study, they show that the disruption of packs, the loss of their territories, and the subsequent early dispersal of juveniles happen in populations experiencing high harvest pressure. Other studies have reported young animals leaving their natal pack already at the age of five months; however, dispersal usually happens at the age of 11 to 24 months [41,42]. Dispersal is driven by both social and food competition, but may also be caused by the disruption of a pack’s social structure [35,42,77,78]. According to the genetic data, juveniles were hunted relatively far from their natal territories already at an age of five to ten months. Currently, in Latvia, the lack of food resources should not be the reason for competition in packs, and before reaching sexual maturity, the young animals do not create social competition in the packs. Therefore, the early dispersal from packs is likely caused by the disruption of the social and territorial structures due to hunting. Early dispersal and the relatively easy availability of breeding opportunities, due to lost breeders and vacated pack territories, create both the opportunity and the necessity for young animals to start breeding at an early age in order to compensate for the numbers of individuals lost due to hunting. In the Latvian wolf population, cases where females started breeding even before two years of age were identified. Also, the majority of reproductively active females were two- to three-year-old animals, and relatively large average litter sizes and a high proportion of females involved in reproduction were found [30]. This indicates heightened reproductive activity in the population. As young breeders may lack experience and knowledge to ensure high reproductive success, and young females tend to have smaller litters [74,79,80], such possible demographic consequences of social instability should not be overlooked considering the long-term effects that hunting might have on population conservation.

A turnover of individuals has also been found in some naturally regulated, unexploited populations and populations experiencing low hunting pressure [3,11,47,48]. However, it usually affects the social structure of the population on a smaller scale and in less disruptive ways than the impact caused by the higher levels of human exploitation. In cases of breeder loss due to natural mortality, pack persistence was higher than when breeder loss was due to anthropogenic mortality [81]. In populations with lower harvest intensity, packs usually kept their territories, although they experienced some turnover of individuals [3,56], while in unexploited populations, individual turnover and acceptance of unrelated animals into packs were very low and kinship within packs was high, indicating a stable, natural social structure [11].

In order to reduce the negative impact of hunting on the stability of the social structure of the population and possible long-term negative consequences, not only the size and distribution of the population should be taken into account when evaluating the annual hunting limit. The impact of hunting on the demographic and social structure of the population, as well as food resources available to predators and the potential future impact of any obstacles to the migration of animals, for example, fences being built on country borders, should also be considered. In addition, when evaluating the duration of the hunting season, the impact of hunting on the social and territorial stability of the population and the possibilities of preserving functional wolf packs should be taken into account.

To improve knowledge about social processes in the Latvian wolf population and its kinship structure, we recommend obtaining genetic material from neighbouring countries in order to clarify wolf kinship and social structure in border areas and the importance of immigrants in maintaining the local population. In addition, the introduction of noninvasive genetic monitoring is advisable to obtain more comprehensive information about ongoing processes within and among wolf packs and to provide a more detailed insight into the social dynamics of the population and the dispersal habits of the individuals. Probable effects of social instability on the demographic structure and reproductive processes in the population as well as on the increase in depredation cases should be investigated in future studies.

## 5. Conclusions

At the time of this study, hunting pressure had not reduced the numbers and distribution of individuals in the Latvian wolf population [30], negative changes were not observed in the genetic parameters, and it had not contributed to the fragmentation of the population. Current hunting practices can be considered sustainable in regard to the abundance and distribution of animals and their ability to compensate for harvested individuals. However, it should be taken into account that populations of any species are not only a natural resource which can be measured in a certain number of animals that are successfully able to reproduce over a long period of time. Every species has certain ecological functions and relevance, and the effects of human activities on the quality of life of animals and social relations among individuals should be taken into account, especially for animals that live in family groups and where a stable social structure is important for the long-term preservation of the species. We observed a negative impact of hunting on the stability of territorial and social structure of the Latvian wolf population in breeder loss, pack disruptions, abandonments of pack territories, the early dispersal of young and inexperienced animals, the frequent turnover of individuals in packs, short-lived kin groups, and lower relatedness levels observed in some parts of the population, which may indicate that at least part of the population depends on immigrants for its sustainability.

It is not expected that the length of the wolf hunting season in Latvia could be changed or the limits reduced in the near future, as the number of wolves has increased in the last decade [30], society is mostly satisfied with the existing management system [82], and hunting is considered a necessary means to reduce depredation, even if its effectiveness in mitigating this conflict remains unclear [83]. Thus, the results of this study can serve as an assessment of the social structure and genetic characteristics of the harvested wolf population and an initial assessment of the negative impact of hunting on these parameters. These findings should be taken into account in future studies of population sustainability and the implementation of conservation measures.

## Figures and Tables

**Table 1:** Longevity of the groups (*n* = 180) of kindred wolves in Latvia as established from kinship analyses of the harvested wolves.

Group Duration (Years)	11	10	9	8	7	6	5	4	3	2	1
Proportion in the sample of kinship analyses (%)	0.6	1.7	1.7	4.4	2.2	3.9	5.0	10.0	8.9	15.6	46.1

## Data Availability

Some of the data presented in this study are available in the Supplementary Material. The remaining data are available upon request from the corresponding author.

## Supplementary Materials

The following supporting information can be downloaded at: https://www.mdpi.com/article/10.3390/biology12091255/s1, Table S1: Genotypes, region, birth year and sex of analyzed wolf individuals; Table S2: Distribution of the collected genetic sample numbers per year; Figure S2.1: Genetic diversity parameters of the Latvian wolf population according to birth year of the individuals; S2.2: Changes in the observed heterozygosity values during the study period; S2.3: Changes in the fixation (inbreeding) index values during the study period.

## References

[1] Taberlet P., Luikart G., Geffen E., Gittleman J.L., Funk S.M., Macdonald D.W., Wayne R.K. (2001). New methods for obtaining and analyzing genetic data from free-ranging carnivores. Carnivore Conservation.

[2] Mills S.L. (2007). Conservation of Wildlife Populations: Demography, Genetics, and Management.

[3] Caniglia R., Fabbri E., Galaverni M., Milanesi P., Randi E. (2014). Noninvasive sampling and genetic variability, pack structure, and dynamics in an expanding wolf population. J. Mammal..

[4] Reinhardt I., Kluth G., Nowak S., Mysłajek R.W. (2015). Standards for the Monitoring of the Central European Wolf Population in Germany and Poland.

[5] Waser P.M., Strobeck C., Paetkau D., Gittleman J.L., Funk S.M., Macdonald D.W., Wayne R.K. (2001). Estimating interpopular dispersal rates. Carnivore Conservation.

[6] Lucchini V., Fabbri E., Marucco F., Ricci S., Boitani L., Randi E. (2002). Noninvasive molecular tracking of colonizing wolf (*Canis lupus*) packs in the western Italian Alps. Mol. Ecol..

[7] Valière N., Fumagalli L., Gielly L., Miquel C., Lequette B., Poulle M.L., Weber J., Arlettaz R., Taberlet P. (2003). Long-distance wolf recolonization of France and Switzerland inferred from non-invasive genetic sampling over a period of 10 years. Anim. Conserv..

[8] Andersen L.W., Harms V., Caniglia R., Czarnomska S.D., Fabbri E., Jędrzejewska B., Kluth G., Madsen A.B., Nowak C., Pertoldi C. (2015). Long-distance dispersal of a wolf, *Canis lupus*, in northwestern Europe. Mammal Res..

[9] Liberg O., Aronson A., Sand H., Wabakken P., Maartmann E., Svensson L., Åkesson M. (2012). Monitoring of wolves in Scandinavia. Hystrix Ital. J. Mammal..

[10] Wayne R.K., Vilà C., Mech L.D., Boitani L. (2003). Molecular genetic studies of wolves. Wolves: Behavior, Ecology and Conservation.

[11] Rutledge L.Y., Patterson B.R., Mills K.J., Loveless K.M., Murray D.L., White B.N. (2010). Protection from harvesting restores the natural social structure of eastern wolf packs. Biol. Conserv..

[12] Rick J.A., Moen R.A., Erb J.D., Strasburg J.L. (2017). Population structure and gene flow in a newly harvested gray wolf (*Canis lupus*) population. Conserv. Genet..

[13] Mysłajek R.W., Tracz M., Tracz M., Tomczak P., Szewczyk M., Niedźwiecka N., Nowak S. (2018). Spatial organization in wolves *Canis lupus* recolonizing north-west Poland: Large territories at low population density. Mamm. Biol..

[14] Pilot M., Greco C., Von Holdt B.M., Jedrzejewska B., Randi E., Jedrzejewski W., Sidorovich V.E., Ostrander E.A., Wayne R.K. (2014). Genome-wide signatures of population bottlenecks and diversifying selection in European wolves. Heredity.

[15] Szewczyk M., Nowak S., Niedźwiecka N., Hulva P., Špinkytė-Bačkaitienė R., Demjanovičová K., Bolfíková B.Č., Antal V., Fenchuk V., Tomczak M.P. (2019). Dynamic range expansion leads to establishment of a new, genetically distinct wolf population in Central Europe. Sci. Rep..

[16] Wayne R.K., Brown D.M., Gittleman J.L., Funk S.M., Macdonald D.W., Wayne R.K. (2001). Hybridization and conservation of carnivores. Carnivore Conservation.

[17] Andersone Z., Lucchini V., Randi E., Ozolins J. (2002). Hybridisation between wolves and dogs in Latvia as documented using mitochondrial and microsatellite DNA markers. Mammal. Biol..

[18] Hindrikson M., Männil P., Ozolins J., Krzywinski A., Saarma U. (2012). Bucking the Trend in Wolf-Dog Hybridization: First Evidence from Europe of Hybridization between Female Dogs and Male Wolves. PLoS ONE.

[19] Stenglein J.L., Waits L.P., Ausband D.E., Zager P., Mack C.M. (2011). Estimating gray wolf pack size and family relationships using noninvasive genetic sampling at rendezvous sites. J. Mammal..

[20] Galaverni M., Palumbo D., Fabbri E., Caniglia R., Greco C., Randi E. (2012). Monitoring wolves (*Canis lupus*) by non-invasive genetics and camera trapping: A small-scale pilot study. Eur. J. Wildl. Res..

[21] Ellegren H. (1999). Inbreeding and relatedness in Scandinavian grey wolves *Canis lupus*. Hereditas.

[22] Gomerčić T., Sindičić M., Galov A., Arbanasić H., Kusak J., Kocijan I., Gomerčić M., Huber Ð. (2010). High genetic variability of the grey wolf (*Canis lupus* L.) population from Croatia as revealed by mitochondrial DNA control region sequences. Zool. Stud..

[23] Shakarashvili M., Kopaliani N., Gurielidze Z., Dekanoidze D., Ninua L., Tarkhnishvili D. (2020). Population genetic structure and dispersal patterns of grey wolf (*Canis lupus*) and golden jackal (*Canis aureus*) in Georgia, the Caucasus. J. Zool..

[24] VonHoldt B.M., Stahler D.R., Smith D.W., Earl D.A., Pollinger J.P., Wayne R.K. (2008). The genealogy and genetic viability of reintroduced Yellowstone grey wolves. Mol. Ecol..

[25] Pilot M., Jedrzejewski W., Branicki W., Sidorovich V.E., Jedrzejewska B., Stachura K., Funk S. (2006). Ecological factors influence population genetic structure of European grey wolves. Mol. Ecol..

[26] Hindrikson M., Remm J., Männil P., Ozolins J., Tammeleht E., Saarma U. (2013). Spatial Genetic Analyses Reveal Cryptic Population Structure and Migration Patterns in a Continuously Harvested Grey Wolf (*Canis lupus*) Population in North-Eastern Europe. PLoS ONE.

[27] Stronen A.V., Jędrzejewska B., Pertoldi C., Demontis D., Randi E., Niedziałkowska M., Pilot M., Sidorovich V.E., Dykyy I., Kusak J. (2013). North-South Differentiation and a Region of High Diversity in European Wolves (*Canis lupus*). PLoS ONE.

[28] Ozoliņš J., Andersone Ž. (2002). Management Plan for Wolf (Canis lupus) in Latvia.

[29] Ozoliņš J., Žunna A., Ornicāns A., Done G., Stepanova A., Pilāte D., Šuba J., Lūkins M., Howlett S.J., Bagrade G. (2017). Action Plan for Grey Wolf Canis lupus Conservation and Management.

[30] Šuba J., Žunna A., Bagrade G., Done G., Lūkins M., Ornicāns A., Pilāte D., Stepanova A., Ozoliņš J. (2021). Closer to Carrying Capacity: Analysis of the Internal Demographic Structure Associated with the Management and Density Dependence of a Controlled Wolf Population in Latvia. Sustainability.

[31] Boitani L., Kaczensky P., Alvares F., Andrén H., Balys V., Blanco J.C., Chapron G., Chiriac S., Cirovic D., Drouet-Houguet N. (2022). Assessment of the Conservation Status of the Wolf (Canis lupus) in Europe.

[32] Anonymous Wolf (*Canis lupus*) Protection Plan. Environment Ministry of the Republic of Lithuania, Vilnius, 2014. http://www.vilkai.lt/wp-content/uploads/LTU_Wolf_Protection_Plan_2014_en.pdf.

[33] Remm J., Hindrikson M. Estonian Conservation and Management Plan of Large Carnivores 2022–2031. Environmental Board, Pärnu, Estonia, 2022. https://keskkonnaamet.ee/en/news/environmental-board-approved-new-action-plan-protection-and-management-large-carnivores-ten.

[34] Jędrzejewski W., Jędrzejewska B., Andersone-Lilley Ž., Balčiauskas L., Männil P., Ozoliņš J., Sidorovich V.E., Bagrade G., Kübarsepp M., Ornicāns A., Musiani M., Boitani L. (2010). Synthesizing wolf ecology and management in Eastern Europe: Similarities and contrasts with North America. The World of Wolves: New Perspectives on Ecology, Behaviour and Management.

[35] Ballard W.B., Whitman J.S., Gardner C.L. (1987). Ecology of an Exploited Wolf Population in South-Central Alaska. Wildl. Monogr..

[36] Fuller T.K., Mech L.D., Cohraine J.F., Mech L.D., Boitani L. (2003). Wolf population dynamics. Wolves: Behavior, Ecology and Conservation.

[37] Adams L.G., Stephenson R.O., Dale B.W., Ahgook R.T., Demma D.J. (2008). Population dynamics and harvest characteristics of wolves in the Central Brooks Range, Alaska. Wildl. Monogr..

[38] Creel S., Rotella J.J. (2010). Meta-Analysis of Relationships between Human Offtake, Total Mortality and Population Dynamics of Gray Wolves (*Canis lupus*). PLoS ONE.

[39] Haber G.C. (1996). Biological, Conservation, and Ethical Implications of Exploiting and Controlling Wolves. Conserv. Biol..

[40] Johnson W.E., Eizirik E., Lento G.M., Gittleman J.L., Funk S.M., Macdonald D.W., Wayne R.K. (2001). The control, exploitation, and conservation of carnivores. Carnivore Conservation.

[41] Mech L.D. (1970). The Wolf: The Ecology and Behaviour of an Endangered Species.

[42] Mech L.D., Boitani L., Mech L.D., Boitani L. (2003). Wolf Social Ecology. Wolves: Behavior, Ecology and Conservation.

[43] Silk J.B. (2007). The adaptive value of sociality in mammalian groups. Philos. Trans. R. Soc. Lond. B Biol. Sci..

[44] Cassidy K.A., MacNulty D.R., Stahler D.R., Smith D.W., Mech L.D. (2015). Group composition effects on aggressive inter-pack interactions of gray wolves in Yellowstone National Park. Behav. Ecol..

[45] Cassidy K.A., McIntyre R.T. (2016). Do gray wolves (*Canis lupus*) support pack mates during aggressive inter-pack interactions?. Anim. Cognit..

[46] Jędrzejewska B., Jędrzejewski W., Bunevich A.N., Miłkowski L., Okarma H. (1996). Population Dynamics of Wolves *Canis lupus* in Białowieża Primeval Forest (Poland and Belarus) in relation to hunting by humans, 1847–1993. Mammal Rev..

[47] Brainerd S.M., Andrén H., Bangs E.E., Bradley E.H., Fontaine J.A., Hall W., Iliopoulos Y., Jimenez M.D., Jozwiak E.A., Liberg O. (2008). The effects of breeder loss on wolves. J. Wildl. Manag..

[48] Ausband D.E., Mitchell M.S., Waits L.P. (2017). Effects of breeder turnover and harvest on group composition and recruitment in a social carnivore. J. Anim. Ecol..

[49] Wallach A.D., Ritchie E.G., Read J., O’Neill A.J. (2009). More than Mere Numbers: The Impact of Lethal Control on the Social Stability of a Top-Order Predator. PLoS ONE.

[50] Jędrzejewski W., Branicki W., Veit C., MeĐugorac I., Pilot M., Bunevich A.N., Jędrzejewska B., Schmidt K., Theuerkauf J., Okarma H. (2005). Genetic diversity and relatedness within packs in an intensely hunted population of wolves *Canis lupus*. Acta Theriol..

[51] Frank L.G., Woodroffe R., Gittleman J.L., Funk S.M., Macdonald D.W., Wayne R.K. (2001). Behaviour of carnivores in exploited and controlled populations. Carnivore Conservation.

[52] Allendorf F.W., England P.R., Luikart G., Ritchie P.A., Ryman N. (2008). Genetic effects of harvest on wild animal populations. Trends Ecol. Evol..

[53] Funk S.M., Fiorello C.V., Cleaveland S., Gompper M.E., Gittleman J.L., Funk S.M., Macdonald D.W., Wayne R.K. (2001). The role of disease in carnivore ecology and conservation. Carnivore Conservation.

[54] Ausband D.E., Waits L. (2020). Does harvest affect genetic diversity in grey wolves?. Mol. Ecol..

[55] Linnell J., Salvatori V., Boitani L. (2008). Guidelines for Population Level Management Plans for Large Carnivores.

[56] Bassing S.B., Ausband D.E., Mitchell M.S., Lukacs P., Keever A., Hale G., Waits L. (2019). Stable pack abundance and distribution in a harvested wolf population. J. Wildl. Manag..

[57] Haig S.M., Ballou J.D., Beissinger S.R., McCullough D.R. (2002). Pedigree Analyses in Wild Populations. Population Viability Analysis.

[58] Klevezal G.A. (1988). Age-Related Structures in Zoological Studies of Mammals.

[59] Francisco L.V., Langsten A.A., Mellersh C.S., Neal C.L., Ostrander E.A. (1996). A class of highly polymorphic tetranucleotide repeats for canine genetic mapping. Mamm. Genome.

[60] Shibuya H., Collins B.K., Huang T.M., Johnson G.S. (1994). A polymorphic (AGGAAT), tandem repeat in an intron of the canine von Willebrand factor gene. Anim. Genet..

[61] Holmes N.G., Dickens H.F., Parker H.L., Binns M.M., Mellersh C.S., Sampson J. (1995). Eighteen canine microsatellites. Anim. Genet..

[62] Fredholm M., Winterø A.K. (1995). Variation of short tandem repeats within and between species belonging to the Canidae family. Mamm. Genome.

[63] Ostrander E.A., Mapa F.A., Yee M., Rine J. (1995). One hundred and one new simple sequence repeat-based markers for the canine genome. Mamm. Genome.

[64] Peakall R., Smouse P.E. (2012). GenAlEx 6.5: Genetic analysis in Excel. Population genetic software for teaching and research-an update. Bioinformatics.

[65] Queller D.C., Goodnight K.F. (1989). Estimating relatedness using genetic markers. Evolution.

[66] Goudet J.F. (1995). FSTAT (version 1.2): A computer program to calculate F-statistics. J. Hered..

[67] Jones O.R., Wang J. (2010). COLONY: A program for parentage and sibship inference from multilocus genotype data. Mol. Ecol. Resour..

[68] Kalinowski S.T., Taper M.L., Marshall T.C. (2007). Revising how the computer program Cervus accommodates genotyping error increases success in paternity assignment. Mol. Ecol..

[69] Ozoliņš J., Stepanova A., Žunna A., Bagrade G., Ornicāns A., Stubbe M. (2011). Wolf hunting in Latvia in the light of population continuity in the Baltics. Beiträge zur Jagd- und Wildforschung.

[70] Baltrūnaité L., Balčiauskas L., Åkesson M. (2013). The genetic structure of the Lithuanian wolf population. Centr. Eur. J. Biol..

[71] Hindrikson M., Remm J., Pilot M., Godinho R., Stronen A.V., Baltrūnaité L., Czarnomska S.D., Leonard J.A., Randi E., Nowak C. (2017). Wolf population genetics in Europe: A systematic review, meta-analysis and suggestions for conservation and management. Biol. Rev..

[72] Palsbøll P.J., Peery M.Z., Bérubé M. (2010). Detecting populations in the ‘ambiguous’ zone: Kinship-based estimation of population structure at low genetic divergence. Mol. Ecol. Resour..

[73] Lehman N., Clarkson P., Mech L.D., Meier T.J., Wayne R.K. (1992). A study of the genetic relationships within and among wolf packs using DNA fingerprinting and mitochondrial DNA. Behav. Ecol. Sociobiol..

[74] Packard J.M., Mech L.D., Boitani L. (2003). Wolf behavior: Reproductive, social, and intelligent. Wolves: Behavior, Ecology and Conservation.

[75] Eklund A., López-Bao J.V., Tourani M., Chapron G., Frank J. (2017). Limited evidence on the effectiveness of interventions to reduce livestock predation by large carnivores. Sci. Rep..

[76] Balčiauskas L., Balčiauskienė L., Litvaitis J.A., Tijušas E. (2020). Adaptive monitoring: Using citizen scientists to track wolf populations when winter-track counts become unreliable. Wildl. Res..

[77] Hayes R.D., Harestad A.S. (2000). Demography of a recovering wolf population in the Yukon. Can. J. Zool..

[78] Fuller T.K., Sievert P.R., Gittleman J.L., Funk S.M., Macdonald D.W., Wayne R.K. (2001). Carnivore demography and the consequences of changes in prey availability. Carnivore Conservation.

[79] Kojola I. (2005). Status and development of the wolf population in Finland. Management Plan for the Wolf Population in Finland.

[80] Mech L.D., Barber-Meyer S.M., Erb J. (2016). Wolf (*Canis lupus*) Generation Time and Proportion of Current Breeding Females by Age. PLoS ONE.

[81] Borg B.L., Brainerd S.M., Meier T.J., Prugh L.R. (2015). Impacts of breeder loss on social structure, reproduction and population growth in a social canid. J. Anim. Ecol..

[82] Žunna A., Bagrade G., Ozoliņš J. (2020). Attitudes of the General Public and Hunters Towards Wolves in Latvia; Its Predictors and Changes Over Time. Proc. Latv. Acad. Sci. Sect. B.

[83] Šuba J., Žunna A., Bagrade G., Done G., Ornicāns A., Pilāte D., Stepanova A., Ozoliņš J. (2023). Does Wolf Management in Latvia Decrease Livestock Depredation? An Analysis of Available Data. Sustainability.

